# Evaluation of Mechanical Versus Manual Root Canal Preparation in Primary Molars—A Comparative In Vitro Study

**DOI:** 10.3390/jcm12247718

**Published:** 2023-12-15

**Authors:** Nelly Schulz-Weidner, Jiameng Wang, Jessica Steinbart, Anton G. Windfelder, Gabriele A. Krombach, Norbert Krämer, Maximiliane A. Schlenz, Jan Scherberich

**Affiliations:** 1 Department of Pediatric Dentistry, Dental Clinic, Justus Liebig University, 35392 Giessen, Germany; nelly.schulz-weidner@dentist.med.uni-giessen.de (N.S.-W.); jiameng.wang@dentist.med.uni-giessen.de (J.W.); norbert.kraemer@dentist.med.uni-giessen.de (N.K.); 2 Experimental Radiology, Department of Diagnostic and Interventional Radiology, University Hospital Giessen, Justus Liebig University Giessen, 35392 Giessen, Germany; jessica.steinbart@radiol.med.uni-giessen.de (J.S.); anton.g.windfelder@radiol.med.uni-giessen.de (A.G.W.); 3 Branch for Bioresources, Fraunhofer Institute for Molecular Biology and Applied Ecology IME, 35392 Giessen, Germany; 4 Department of Diagnostic and Interventional Radiology, University Hospital Giessen, Justus Liebig University Giessen, Giessen 35392, Germany; gabriele.krombach@radiol.med.uni-giessen.de; 5 Department of Prosthodontics, Dental Clinic, Justus Liebig University, 35392 Giessen, Germany; maximiliane.a.schlenz@dentist.med.uni-giessen.de

**Keywords:** dentistry, pediatric dentistry, endodontics, primary teeth, root canal preparation, micro-computed tomography, comparative study, diagnostic imaging

## Abstract

The endodontic treatment of primary teeth is to maintain the function of the tooth free of symptoms until its physiological exfoliation. A critical factor for success is how quickly and effectively the root canal preparation can be performed. Therefore, the aim of this comparative in vitro study was to analyze the efficiency of two mechanical root canal preparation systems FM (FlexMaster) and HF (HyFlex EDM) to manual KF (K-file) on extracted primary molars. A total of 45 teeth were divided into three groups (*n* = 15): KF (#15–35), FM (04#30) and HF (25/~ OneFile). Root canal preparation was performed, and the preparation time was measured. All root canals were non-destructively analyzed by micro-computed tomography in the cervical, middle and apical thirds before and after preparation with regard to the parameters of canal transport (in μm) and centering ratio (0–1). Statistical analysis was performed at a 5% significance level using non-parametric tests. HF caused the lowest canal transport in the apical third (*p =* 0.008). The centering ratio value of HF was significantly higher in the middle third of the root canals than in the other two groups (*p* < 0.01). The mean instrumentation time was significantly higher for KF (6.67 min) than for FM (4.69 min) and HF (4.03 min, *p* < 0.01). HF can be recommended for primary molar root canal treatment.

## 1. Introduction

Data from epidemiological studies indicate that the prevalence of caries in primary dentition continues to be high [1]. Due to anatomical and biological factors, carious lesions in primary teeth quickly lead to inflammation of the pulp, or the pulp often opens when excavating and preparing the cavity [2]. As a result, pulp diseases are not uncommon in the primary dentition. Due to the value of the second primary molars for the proper eruption of the permanent teeth, as well as for chewing function, in particular, the preservation of these teeth plays an important role. For this reason, second primary molars should be retained until the first permanent molars are fully erupted, if possible, to prevent the gap from narrowing or even closing [3].

One of the endodontic treatment methods for primary teeth is pulpectomy followed by preparation and filling [4]. Because of the complicated morphology of the root canals, especially in primary molars and the lack of the cooperation of the child during the treatment, the treatment of primary teeth can be complex. Fast and effective root canal preparation creates the conditions for subsequent root canal filling and is, therefore, one of the keys to successful root canal treatment in primary molars.

Not only the materials used but also the instrumentation techniques have improved in the field of pulp therapy in recent years. Technological improvements, such as machine preparation, lead to higher-quality work in less time [5]. However, the gold standard in pediatric dentistry is still manual preparation with K-files (KF, Kerr, Glendora, CA, USA) [4,5,6,7,8], as they have the highest cutting performance and best shape of all handheld rotary instruments [9]. Therefore, K-files were selected as the control group. FlexMaster^®^ (FM, VDW, Munich, Germany) is the third generation of nickel-titanium (NiTi) instruments and utilizes the efficiency of the proven ‘K’ cutting edges, a convex cross-section to stabilize the instrument core, and a cutting-edge inclination adapted to the rotary application [10]. This design is thought to improve the efficiency of cutting dentin with rotary instruments, although more aggressive cutting may result in increased canal transport [9]. FlexMaster instruments are available with tapers of 0.02, 0.04 and 0.06 and in sizes from 20 to 45. These higher-tapered instruments were introduced to optimize canal shape, reduce the incidence of instrument failure, and improve the comparatively poor cutting performance of nickel–titanium instruments [10]. The new HyFlex^®^ Electrical Discharge Machining (EDM) files (HF, OneFile, Coltène, Altstätten, Switzerland) represent the fifth generation of root canal files. HyFlex EDM files have a unique surface due to their novel EDM process, making them stronger and more resistant to fracture. According to the manufacturer, Hyflex EDM files exhibit a crystallographic phase change from austenite to martensite at room temperature, unlike commercially available NiTi files, making them highly flexible and fracture-resistant. Due to the Controlled Memory Effect (controlled restoring force), the files always work in the center of the canal, which significantly reduces the risk of via falsa, canal center displacement, or canal perforation. The analysis of a systemic review showed that the use of martensitic endodontic instruments results in less apical transport during the endodontic shaping phases compared with austenitic instruments, due to the greater flexibility of martensitic instruments [11]. By using HyFlex EDM files, the number of files can be reduced to 2–3, depending on the clinical situation, especially in straight and larger canals.

Panchal et al. summarized the main methods used to evaluate cleaning efficiency in in vitro studies, including the verification of ink removal from canals, smear layer removal, pre- and post-imaging with stereo microscopy, cone beam computed tomography and spiral computed tomography [12]. New applications of micro-computed tomography (μ-CT) in endodontic research have resulted from improvements in scanning technology and imaging software. μ-CT is a noninvasive, nondestructive, high-resolution technology that allows a three-dimensional examination of the root canal system [10,13]. With this technology, the influence of various treatments on the root canals can be tracked by digitally reconstructing tooth cross-sections that can be assembled into three-dimensional models. After reconstructing 3D models and cross-sections of the root canals from μ-CT scans, Fumes et al. analyzed the primary tooth samples [14]. Hidalgo et al. scanned the primary molars before and after root canal preparation and reconstructed the preoperative and postoperative μ-CT images to evaluate the changes in 2D and 3D morphological parameters, as well as canal transport and lateral perforation [6]. Barasuol et al. examined the primary molar specimens before and after chemical–mechanical preparation using μ-CT to compare canal transport and the percentage of dentin removed [15].

Modern mechanical files in root canal treatment offer clinical advantages by efficiently shaping the canal, reducing treatment time [12]. Moreover, it could minimize procedural errors, fatigue, and provide more consistent and precise outcomes compared to manual files, enhancing overall patient care and treatment success rates in complex cases.

Thus, the aim of this study is to compare the efficiency of two mechanical root canal preparation systems (FM and HF) with manual KF on extracted primary molars using μ-CT technology for evaluation. It was hypothesized that there is no significant difference between the mechanical file systems (FM and HF) and KF in terms of root canal transport, centering ratio and instrumentation time.

## 2. Materials and Methods

### 2.1. Study Design

In accordance with the approval of the Ethics Committee of the Medical Faculty of the Justus Liebig University of Giessen (ref. no. 143/09), a comparative in vitro study was designed. Therefore, extracted primary molars, not worth preserving due to deep carious lesions, were collected, cleaned with scalers/curettes and stored in 0.5% chloramine-T solution for a maximum of four weeks. If the teeth could not be used immediately, they were frozen in distilled water at −21 °C.

The inclusion criteria for root canals were defined as [5,16]:

- Distal, palatal, mesial and buccal roots of primary molars with a length of ≥7 mm;
- Radii > 5 mm.

The following exclusion criteria were established:

- Resorption in the cervical or middle third of the root canals;
- Root caries;
- Previous endodontic treatment;
- Abnormality in tooth morphology.

A total of 45 primary molars were randomized to three study groups (*n* = 15 teeth per group with three root canal preparations per tooth, 3 × 15 = 45 root canal preparations per study group):

- FlexMaster^®^ (FM, 04 #30, VDW);
- Hyflex^®^ EDM NiTi files (HF, 25/~ OneFile, Coltène);
- K-Files (KF, #15–35, Kerr).

### 2.2. Preliminary Tests

All root canal preparations were conducted by the same dentist (J.W.), who has a postgraduate specialization in pediatric dentistry. Prior to this study, J.W. was trained by a specialist in pediatric dentistry (N.S.-W.) with over 20 years of experience. In addition, J.W. had undertaken extensive pre-testing of the various instruments. Theoretical and practical knowledge of the three file systems was gained by reading the manufacturer’s instructions for use and performing a preliminary test. The latter involved the root canal preparation of five teeth per system, which were not included in the present study.

### 2.3. Root Canal Preparation

Before root canal preparation, the root length was passively measured to the apical foramen with a #10 K-file and then shortened by 1 mm. After the determination of the length, the following preparation techniques were used:

- FM (experimental group 1):
- Root canals were mechanically prepared with the established Crown-Down NiTi System FM system, according to the manufacturer’s instructions. The last file used was size 04 #30.
- HF (experimental group 2):
- After creating a straight coronal access, the Hyflex CM 25/0.08 file was used as an orifice opener. For the mechanical preparation of the root canals with HF, the following files were used according to the manufacturer’s instructions: 10/0.05 Glidepath File, 20/~ Hyflex EDM One File and 25/~ Hyflex EDM One File.
- KF (control group):

The conventional rotary technique was used to manually prepare the root canals with KF. Mechanical cleaning to the calculated root length was performed with KF in the order indicated: ISO-15, ISO-20, ISO-25, ISO-30 and ISO-35.

The same rinsing protocol was used for all study groups: after each endodontic file, rinsing was performed with 2 mL of 1% sodium hypochlorite solution (Histolith, lege artis Pharma GmbH, Dettenhausen, Germany). After root canal preparation, rinsing was performed with 5 mL of 17% EDTA solution (Produits Dentaires SA, Vevey, Switzerland) followed by 5 mL of 1% sodium hypochlorite solution. The root canals were then dried with paper points. In addition, the instrumentation time was recorded with a stopwatch. The time from the insertion of the first endodontic file into the root canal (starting point) to the last rinse with 1% sodium hypochlorite solution (end point) was measured.

Each instrument was used only three times to avoid the deformation or breakage of the endodontic files.

### 2.4. μ-CT Analysis

**Figure 1:**
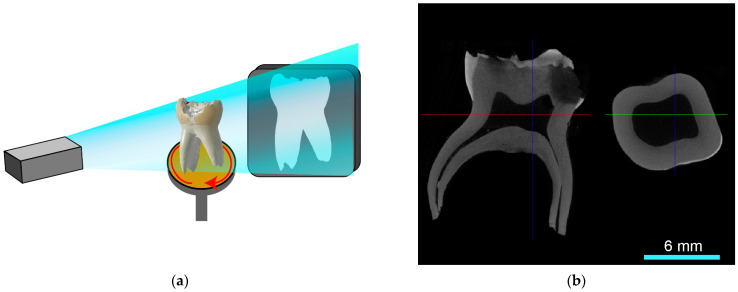
(**a**) In μ-CT measurements, probes are rotated. The electron beam is attenuated by the sample and presented on a detector panel. (**b**) Exemplary μ-CT reconstruction of a primary molar in coronal and transverse plane (scale of 6 mm). The right section of the image shows the transverse plane indicated by the red line.

**Figure 2:**
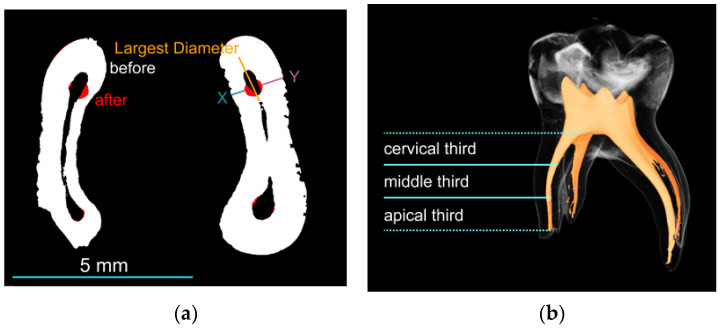
(**a**) Cross-section of a reconstructed tooth scan; the canal transportation and the centering ratio before and after treatment are calculated on the measuring line (blue and purple). Changes due to the treatment are marked in red (scale of 5 mm). (**b**) Rendering of a primary molar; each root canal was divided into cervical, middle and apical thirds.

The measurement of the μ-CT analysis was performed by second operators (J.S., J.S.T.). The primary molars were scanned using X-ray micro-computed tomography (μ-CT, SkyScan 1272, version 1.5.1.0, Bruker, Kontich, Belgium; Figure 1a,b) before and after root canal preparation. For measurement, each root canal was divided into cervical, middle and apical thirds (Figure 2a).

The system contains a 40–100 kV X-ray tube and is equipped with a sCMOS camera. The spot size is <5 μm. The maximum spatial resolution is 0.45 μm (10% modulation transfer function). Projection images were captured per sample on a 16-bit 4096 × 4096 detector. All scans were performed with the same settings at 90 kV (Al 0.5 + Cu 0.038 filter), 111 μA, and an exposure time of 1650 ms. A voxel size of 10 μm was achieved using custom 3D-printed holders. The samples were scanned with a 180° scan in 0.3° steps and the image noise was reduced by a threefold averaging.

The reconstruction from projection images into 8-bit cross-sectional images was carried out with the software NRecon (NRecon 1.7.5.0, Bruker Belgium) with a smoothing parameter of 2, beam hardening of 15% and a ring artifact correction of 8. A dynamic range of 0 to 0.07 was chosen for all reconstructions. If necessary, XY shifts were corrected using reference scans.

The DATAVIEWER^®^ program (DATAVIEWER^®^ 1.7.0.1, © Bruker) was used to visualize the root morphology. For analysis, root canals were calculated using CTan^®^ software (CTan^®^ v1.20.8.0, © 2003–11 SkyScan, © 2012–20Bruker, Kontich, Belgium) and ImageJ (ImageJ 1.54d, Java 1.8.0_345, Wayne Rasband and contributors, National Institutes of Health, MD, USA) and the following formula [17]:

Canal transportation = |(X2 − X1) − (Y2 − Y1)|

Centering ratio = (X2 − X1)/(Y2 − Y1) if (Y2 − Y1) > (X2 − X1)

Or (Y2 − Y1)/(X2 − X1) if (X2 − X1) > (Y2 − Y1)

X1 is the shortest distance from the mesial margin to the periphery of the uninstrumented canal; X2 is the same distance in the corresponding instrumented canal; Y1 is the shortest distance from the distal margin to the periphery of the uninstrumented canal; and Y2 is the same distance in the corresponding instrumented canal (Figure 2a).

Higher values indicate more canal transportation, negative values indicate mesial transport, positive values indicate distal transport, and 0 indicates no transport. The results of this study were compared using the absolute value of root canal transport, considering only the amount of transport and not the direction of transport.

Axial centration indicates the ability of the instrument to maintain the center of the root canal during cutting. A score of 1 indicates perfect centration, while a score close to 0 indicates poor centration (Figure 2a,b).

### 2.5. Statistics

Statistical analysis included one-way ANOVA and post hoc multiple comparisons (least significant difference (LSD)). The Kruskal–Wallis test and Mann–Whitney pairwise test was used for non-normal distributed (Shapiro–Wilk test) data. A 5% significance level was used for all analyses.

## 3. Results

### 3.1. Root Canal Transport

Table 1 displays the results of the root canal transport of the three study groups. All groups showed canal transport. While FM exhibited the highest canal transport in the cervical and middle third, KF showed the highest values in the apical third. No significant difference was observed between the three groups in the cervical and middle third, whereas HF caused less canal transport in the apical third (*p* > 0.05). A high variability of the data was observed.

### 3.2. Centering Ratio

All groups revealed centering ratios < 1, indicating a deviation of the root canal center after instrumentation. A high variability of the data was observed. HF exhibited the highest centering ratio in all thirds of root canal, with no significant difference in the cervical and apical thirds (*p* > 0.05), and a significant difference in the middle third (*p =* 0.008, Table 2).

**Figure 3:**
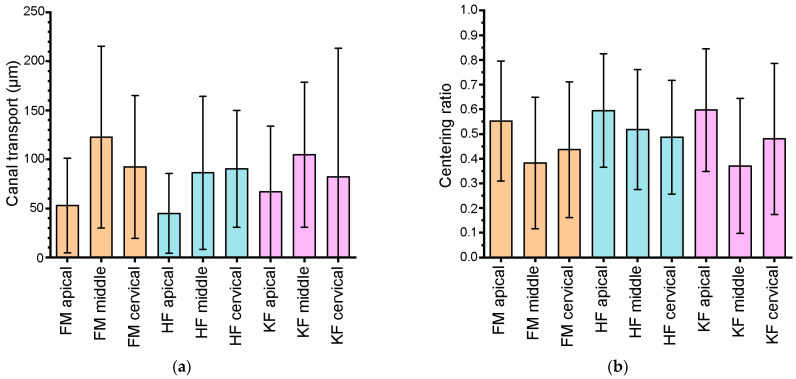
Histogram after preparation of root canals using different instruments: (**a**) canal transport; (**b**) centering ratio; FM: Flexmaster^®^; HF: HyFlex^®^ EDM NiTi files; KF: K-Files.

Figure 3 displays the canal transport (a) and centering ratio (b) for all used instruments after root canal preparation. Whereas FM and KF revealed the highest canal transport in the middle root canal, HF showed the highest value in the cervical root canal. The centering ratio presented for all three types of files in the apical root was the third-highest amount.

### 3.3. Instrumentation Time

**Figure 4:**
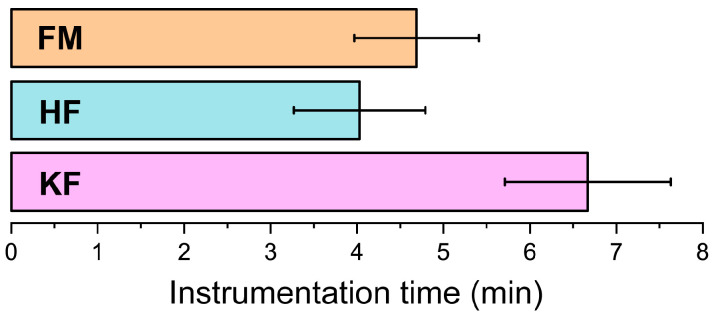
Mean time of instrumentation for the three groups in minutes (min); FM: Flexmaster^®^; HF: HyFlex^®^ EDM NiTi files; KF: K-Files.

The mean instrumentation time was significantly higher in the KF group (6.67 ± 0.96 min) than in the FM (4.69 ± 0.72 min) and HF (4.03 ± 0.76 min) groups (*p* < 0.001, Figure 4).

## 4. Discussion

The null hypothesis that there is no significant difference between the mechanical file systems (FM and HF) and KF in terms of root canal transport, centering ratio and instrumentation time, had to be partially rejected. With regard to the apical third, it was found that manual preparation with KF achieved highest transport, while mechanical preparation with HF performed best. HF also achieved the best values in terms of the centering ratio. Moreover, the use of KF resulted in longer instrumentation times.

The study of human teeth is an established method for root canal instrumentation studies. Its advantages include the hardness, morphology, color, texture and radiopacity of the biological tissue, but there are also some disadvantages: difficulty of extraction, ethical considerations, potential risk of cross-infection, storage and standardization [9]. However, there are also studies that have used simulated resin canals [14,17,18]. Simulated root canals can be used to standardize the diameter of the root canal, the length of the root canal and the radius of curvature [18]. Due to the different hardness or material composition of resin and root dentin, the use of simulated canals in resin blocks does not reflect the results of instruments in root canals of real teeth [10,18]. 

In all three study groups, root canal preparation was performed by the same dentist. This ensured the reliability of the test by avoiding errors caused by different subjective factors when multiple dentists perform an examination. The μ-CT scans were performed by a second operator, who was unaware of the grouping of the samples. Operators 1 and 2 worked together to analyze the image data.

μ-CTs were performed to compare the capabilities of the different instruments. According to the literature [19], a scanning isotropic resolution of 34 and 68 μm is sufficient to analyze the morphology of the root canal, which was ensured in this study by a scanning resolution of 10 μm.

Regarding root canal transport, i.e., the deviation of the root canal preparation instrument from the central axis of the root canal and its excessive incision into the canal wall, the K-files showed the greatest root canal movement in the apical third of the root, which is in accordance with the findings of Barasuol et al. [15]. The authors considered that this could be related to the size of the last file (size #35) used to reach the working length: the reason seems to be the flatness of the apical third of the root, as well as the lower elasticity of the stainless-steel alloy compared with the nickel–titanium file, so that the prepared root canals do not maintain their original orientation. The mechanical properties of the material and the cross-sectional shape of the file might have an influence on root canal transport [11,20,21]. In addition, other factors such as the type of application, the anatomy of the root canal system and the size of the instrument can also influence root canal transport during instrumentation [20]. More aggressive cutting also leads to increased root canal transport [10].

Venino et al. reported that the HF system achieved good results in terms of canal transport, especially in the apical third [21]. The median values ranged from 57 to 129 μm in permanent teeth. This is similar to the findings of the present study. An investigation by Schäfer et al. showed that FM files caused slight canal transport to the outside of the curvature in the apical region of the canals. The NiTi instruments are stiffer than ISO instruments. Therefore, this canal transport can be attributed to root canal preparation with instruments with a greater taper. In contrast, manually prepared canals with KF showed greater transport to the outside of the curvature [22]. 

A deviation from the central axis of more than 0.3 mm was considered clinically significant to affect the apical root fillings and thus influence the clinical outcome [23]. Based on the available results, none of the endodontic files showed transport of more than 0.3 mm, which means that the three file systems are unlikely to cause deviations that affect the clinical outcome. Thus, all three systems were clinically safe for apical root canal preparation.

Centering ability refers to the ability of the axis of the endodontic files to coincide with the axis of the canal during preparation without causing the occlusion, ledging or perforation of the canal [23]. The results of this study show that the centering ratio of the HF files is on average higher than that of the two other groups, although the difference is only significant in the middle third of the root canal (*p* < 0.01). These observations are partially consistent with a previous study by Venino et al. comparing the moldability of ProTaper Next and HF. In this previous study, it was found that the HF instruments performed better in the middle and coronal thirds in terms of the centering ratio [21]. Huang et al. also compared the formability of rotating NiTi systems, including ProTaper Next, HyFlex CM and HyFlex EDM, during root canal preparation in simulated root canals. In their study, HF was found to be better centered than ProTaper Next from 2 mm to 5 mm from the apex, although the differences were only statistically significant at 2 mm and 3 mm from the apex [23]. 

Instrumentation time is shorter with FM and HF than with the KF. The KF has to be changed during the procedure, which takes more time. Previous studies on primary teeth have shown similar results [7,15].

Regarding the selection of the primary molars, the teeth were not classified according to the angle of the root curvature, which needs to be discussed and should be seen as a limitation. 

However, the results show the superiority of mechanical root canal preparation for primary molars. Further studies should now examine whether this is promising in combination with root-filling materials. Enabling faster treatments (less instrumentation time) and easier handling throughout mechanical root canal preparation would be favorable in pediatric dentistry, especially for patients with limited cooperation.

Besides this, in addition to root canal treatment analysis, the high resolution of micro-CT opens up many other evaluation possibilities, including 3D printing.

## 5. Conclusions

All root canal preparation systems differed in terms of canal transport, centering ratio and instrumentation time, with HF being superior to the other two systems in terms of centering ratio, which appears to be important in avoiding via falsa. The instrumentation times of HF and FM are significantly shorter than KF, which also favors the use of mechanical files, especially in pediatric dentistry. Therefore, based on the results, the HF mechanical root canal preparation system can be recommended for primary molar root canal treatment.

## Figures and Tables

**Table 1:** Mean and standard deviation (SD) of canal transport in the cervical, middle and apical thirds of the root canal.

	Cervical Third ^1^ Mean (SD) (μm)	Middle Third ^2^ Mean (SD) (μm)	Apical Third ^3^ Mean (SD) (μm)
FM	80.98 (66.30)	122.58 (92.64)	53.00 (48.13)
HF	85.02 (61.27)	88.13 (80.60)	39.91 (36.86)
KF	69.44 (72.12)	109.11 (81.85)	67.42 (67.69)

^1^ Kruskal-Wallis (*p* = 0.209), Mann–Whitney pairwise (*p* > 0.05). ^2^ Kruskal–Wallis (*p* = 0.163), Mann–Whitney pairwise (*p* > 0.05). ^3^ Kruskal–Wallis (*p* = 0.293), Mann–Whitney pairwise (*p* > 0.05). FM: Flexmaster^®^; HF: HyFlex^®^ EDM NiTi files; KF: K-Files.

**Table 2:** Mean and standard deviation (SD) of the centering ratio in the cervical, middle and apical thirds of the root canal.

	Cervical Third ^1^ Mean (SD) (%)	Middle Third ^2^ Mean (SD) (%)	Apical Third ^3^ Mean (SD) (%)
FM	43.48 (27.16)	38.64 (26.51)	55.96 (24.97)
HF	49.91 (23.67)	51.49 (24.97)	61.59 (22.87)
KF	47.97 (30.93)	37.40 (27.50)	60.00 (24.98)

^1^ Kruskal-Wallis (*p =* 0.547), Mann–Whitney pairwise (*p* > 0.05). ^2^ Kruskal–Wallis (*p* = 0.009), Mann–Whitney pairwise (*p* = 0.008 between KF and HF; *p* = 0.008 between FM and HF). ^3^ ANOVA (*p* = 0.527); post hoc tests (LSD) (*p* > 0.05). FM: Flexmaster^®^; HF: HyFlex^®^ EDM NiTi files; KF: K-Files.

## Data Availability

Data sheets are available at OSF (DOI: 10.17605/OSF.IO/KZUM8). Raw scan data are available upon request from the corresponding author.
